# The kappa/lambda ratio of surface immunoglobulin light chain as a valuable parameter for MRD assessment in CLL with atypical immunophenotype

**DOI:** 10.1038/s41598-024-64398-6

**Published:** 2024-06-11

**Authors:** Yu Aruga, Chiaki Ikeda, Hiromichi Matsushita, Shinichi Makita, Suguru Fukuhara, Wataru Munakata, Koji Izutsu, Hirotaka Matsui

**Affiliations:** 1 Department of Laboratory Medicine, National Cancer Center Hospital, 5-1-1 Tsukiji, Chuo-ku, Tokyo, 104-0045 Japan; 2 Department of Medical Oncology and Translational Research, Graduate School of Medical Sciences, Kumamoto University, Kumamoto, Japan; 3 Department of Laboratory Medicine, Keio University School of Medicine, Tokyo, Japan; 4 Department of Hematology, National Cancer Center Hospital, Tokyo, Japan

**Keywords:** Chronic lymphocytic leukaemia, Leukaemia

## Abstract

In recent years, the significance of detecting minimal/measurable residual disease (MRD) in chronic lymphocytic leukemia (CLL) has increased due to the availability of highly effective therapeutic agents. Flow cytometry provides notable cost-effectiveness and immediacy, with an expected sensitivity level of approximately 10^−4^. The critical aspect of MRD detection via flow cytometry lies in accurately defining the region containing tumor cells. However, a subset of CLL, known as CLL with atypical immunophenotype, exhibits a distinct cell surface marker expression pattern that can make MRD detection challenging, because these markers often resemble those of normal B cells. To enhance the sensitivity of MRD detection in such atypical cases of CLL, we have capitalized on the observation that cell surface immunoglobulin (sIg) light chains tend to be expressed at a higher level in this subtype. For every four two-dimensional plots of cell surface markers, we used a plot to evaluate the expression of sIg kappa/lambda light chains and identified regions where the kappa/lambda ratio of sIg light chains deviated from a designated threshold within the putative CLL cell region. Using this method, we could detect atypical CLL cells at a level of 10^−4^. We propose this method as an effective MRD assay.

## Introduction

Chronic lymphocytic leukemia/small lymphocytic lymphoma (hereafter referred to as CLL) is a lymphoproliferative disorder belonging to the category of mature B-cell malignancies^1,2^. Recent advancements in CLL therapy include molecular targeted agents such as Bruton-type tyrosine kinase inhibitors, and venetoclax, an agent that selectively inhibits BCL-2^3–5^. The introduction of these agents have rendered it feasible for tumor cells in peripheral blood or bone marrow to attain the lower limit of detection, known as minimal/measurable residual disease (MRD), or even fewer^6^.

MRD is generally defined as the presence of fewer than 1 CLL cell in 10,000 cells, detectable in peripheral blood or bone marrow using highly sensitive analytical methods^7^. This can be assessed through a variety of techniques, including flow cytometry, allele-specific oligonucleotide quantitative polymerase chain reaction (PCR), droplet digital PCR, next-generation sequencing (NGS), and cell-free DNA analysis, each having different levels of sensitivity and specificity^8,9^. Clinical studies with molecular targeted therapies have consistently demonstrated that patients with low MRD levels at the end of the administration exhibit improved outcomes in terms of progression-free survival (PFS) and overall survival compared to patients with high MRD levels^10,11^. Additionally, observations suggest that patients with MRD conversion during follow-up tend to experience later disease progression, suggesting the potential utility of regular MRD measurements after treatment completion. However, a recent study evaluating MRD using an NGS-based method reported that the presence or absence of MRD may or may not correlate with PFS, depending on the therapeutic agent used, although the relatively short observation period of the study requires caution in drawing conclusions^12^. Collectively, the significance of MRD measurement in molecular targeted therapies remains uncertain and contentious^13,14^, leading to limited adoption of MRD measurement into routine clinical practice. Nevertheless, the profound response achieved with venetoclax has facilitated the conduct of clinical trials to evaluate the MRD-directed therapy using combinations of venetoclax and other agents^15^. It is anticipated that the outcomes of such clinical studies will provide clarity regarding the utility and positioning of MRD measurements in the future.

The assessment of MRD by detecting tumor cell-specific surface markers using flow cytometry is a commonly used primary assay, and a consensus on this assay method has been achieved through many studies^16,17^. Normal B lymphocytes exhibit a cell surface marker pattern characterized by CD5^−^/CD19^+^/CD20^+^/CD43^−^/CD79b^+^/CD81^+^, whereas CLL cells typically have a marker profile of CD5^dim^/CD19^dim^/CD20^dim^/CD43^dim^/CD79b^−^/CD81^dim^^2,16^. However, there is a certain frequency of different CLL subtypes, referred to as CLL with atypical immunophenotype (hereafter indicated as atypical CLL), characterized by varying surface marker expressions distinct from that of CLL with typical immunophenotype (typical CLL)^17–19^. Standard flow cytometry measurement methods for these cases have not been fully established, and their classification in the disease context remains a topic of debate^18,20–23^. It has also been suggested that atypical CLL may be more prevalent in patients of Asian ethnicity^24,25^, although this has not been extensively studied, and treatment response and prognosis may also differ from typical CLL^26^. Although many of the major clinical studies that have examined the association between molecular targeted therapy and MRD to date have not clearly described atypical CLL, accurately detecting MRD in atypical CLL can be challenging due to similarities in cell surface markers with normal B cells. The paper by the European Research Initiative on CLL (ERIC) that established a standardized MRD detection method underscores this point^27^. It distinguishes between typical CLL, where MRD can be detected without pre-treatment flow cytometry information, and atypical CLL, where pre-treatment flow cytometry information is necessary to detect tumor cells at MRD levels. This underscores the complexity and challenges associated with MRD assessment in this particular subtype of CLL. As a result, it is difficult to determine the response to therapy or predict the prognosis in these cases.

To address this challenge, we have attempted to establish a system for MRD measurement, especially for atypical CLL, by examining the bias of cell surface immunoglobulin (sIg) light chains toward the kappa or lambda type. Although it is currently challenging to automatically determine the appropriate gating for identifying trace amounts of residual disease by flow cytometry, the sensitivity of detection has been enhanced by experimenting with various gate positions and sizes, which can vary from case to case, to identify cell populations with a biased sIg light chain distribution.

## Results

### Distinguishing CLL cells from normal B lymphocytes in atypical CLL poses a significant challenge

We first validated the surface marker expression patterns of CLL cells in our retrospective cohort. A panel of seven antibodies targeting CD5, CD19, CD20, CD43, CD45, CD79b, and CD81, alongside separate tubes for staining sIg light chains within CD19-positive cell populations and staining CD22 and CD23 within CD19-positive cell populations, was utilized (Table 1).

**Table 1:** Antibodies used in the retrospective analysis.

Tube	Antibody	Fluorescence	Clone	Vendor	Amount used (μL)
1	CD43	FITC	1G10	BD	5
	CD81	PE	JS-81	BD	5
	CD5	PECy7	L17F12	BD	1.25
	CD79b	APC	SN8	BD	1.25
	CD19	APCH7	SJ25C1	BD	1.25
	CD20	V450	L27	BD	1.25
	CD45	V500C	2D1	BD	1.25
2	sIg kappa-type light chain	FITC	Rabbit Polyclonal F(ab')2/	DAKO	2.5
	sIg lambda-type light chain	PE			
	CD19	APCH7	SJ25C1	BD	1.25
3	CD22	PE	S-HCL-1	BD	5
	CD23	PECy7	M-L233	BD	1.25
	CD19	APCH7	SJ25C1	BD	1.25

sIg: surface immunoglobulin.

**Figure 1:**
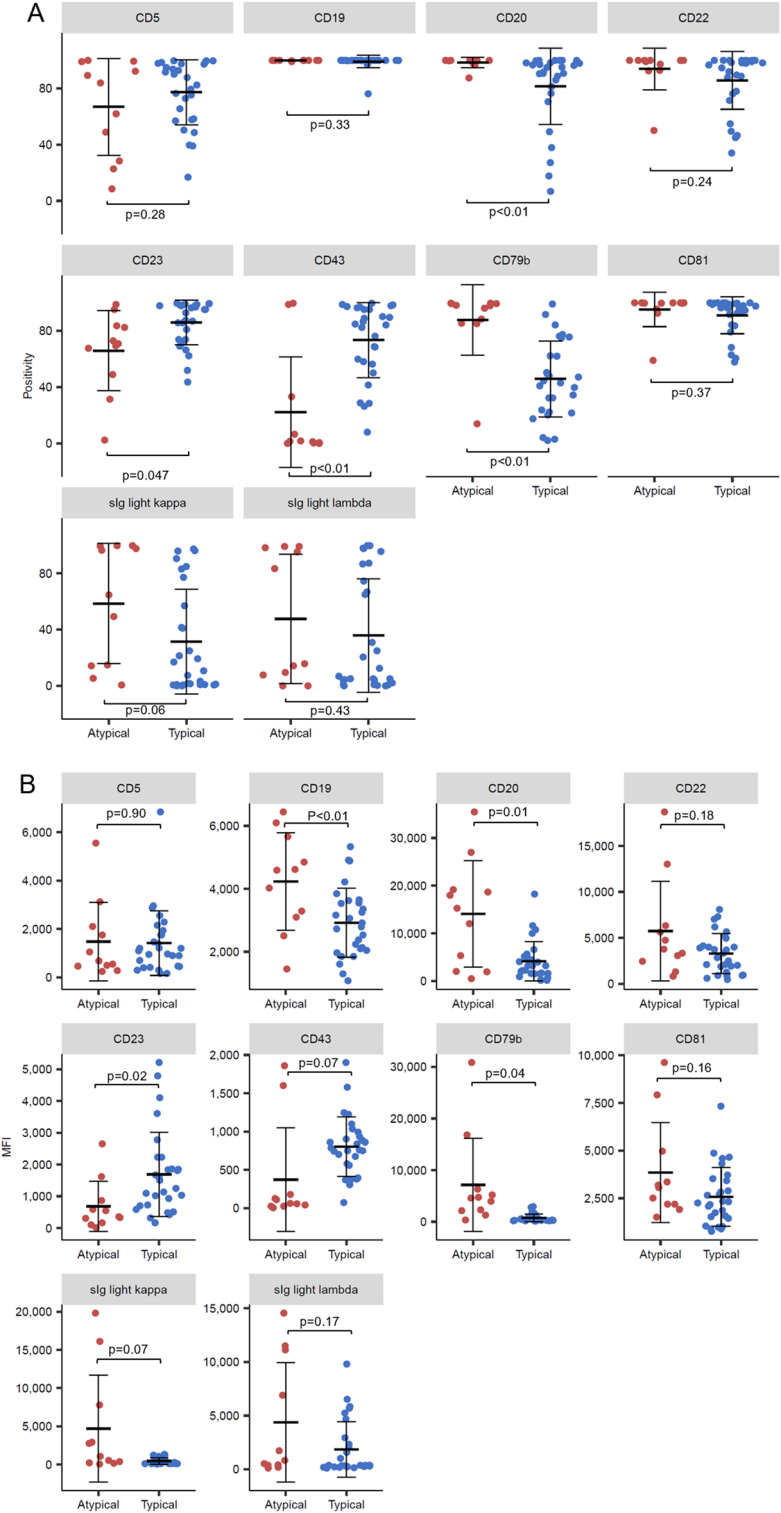
Cell surface marker distributions of CLL and CLL classification in the retrospective analysis. (**A** and **B**) Positivity (**A**) and MFI (**B**) of cell surface markers divided into atypical CLL and typical CLL groups (11 cases and 28 cases, respectively). P-values were calculated between the groups using Welch's *t*-test or Student's *t*-test, depending on whether the values exhibited unequal or equal variances, respectively. A p-value less than 0.05 was considered statistically significant.

The distribution of cell surface markers, including the positivity and mean fluorescence intensity (MFI), within the CD19-positive B cells of the 39 CLL cases is shown in Fig. S1A and B. The results reveal substantial variation in the distribution of surface markers including CD5, CD43, and CD79b among cases, suggesting the inclusion of a considerable number of CLL cases that do not exhibit typical surface markers for CLL in the cohort. Given this, we classified the cases as typical or atypical CLL based on surface markers, with criteria indicated in Materials and methods. The results revealed that our cohort consisted of 28 cases of typical CLL and 11 cases of atypical CLL, which aligns with earlier findings of atypical CLL being more common in Asian descent^25,28,29^. When we compared the distribution of surface markers between the typical and atypical CLL groups, we found that atypical CLL cases had significantly higher positivity for CD20 and lower positivity for CD43 (Fig. 1A). Additionally, when assessed by the MFI, CD19 and CD20 exhibited significantly higher expression. sIg light chains tended to have higher expression, whereas CD43 tended to have lower expression in the atypical CLL group, although these did not reach statistical significance (Fig. 1B). All of these observations are in accordance with previous reports characterizing atypical CLL^21,30^, affirming the accuracy of our typical/atypical classification. These results also suggest that in cases of atypical CLL, distinguishing CLL cells from normal B lymphocytes becomes challenging especially when there are only a few tumor cells, and when normal B lymphocytes have sufficiently recovered from transient decreases resulting from therapies. Indeed, when we established a gating region for CLL cells in each of the 15 cases of atypical CLL and applied the same gate to 12 non-CLL specimens, we discovered that a substantial number of normal B cells fell within the gate in at least three cases of CLL (Fig. S1C). Therefore, further refinement for MRD detection in atypical CLL is deemed necessary. Moreover, due to the absence of a universal gating method for atypical CLL, tailored gating based on individual surface marker expression patterns is warranted.

### Establishment of an MRD detection method applicable to atypical CLL

To address the challenge of distinguishing CLL cells from normal B cells, especially in cases of atypical CLL, based solely on cell surface markers, we established a method for MRD detection in CLL by assessing the bias of sIg kappa/lambda light chains. The process involves staining samples with 12 antibodies in one tube, as described in the Materials and methods (Table 2). The lymphoid-cell region was selected on the FSC-A × SSC-A plot, and within this region, CD19-positive cells were expanded further using combinations of CD22 × CD20, CD43 × CD81, CD79b × CD200, and ROR1 × CD23 markers. Additionally, a development plot was created for each of the four plots to visualize the distribution patterns of sIg kappa/lambda light chains. Appropriate gates, aiming to encompass CLL cells at the highest possible rate, were empirically determined by observing the kappa/lambda ratio within cells that met all gate conditions. An abnormal distribution was defined when a kappa/lambda ratio exceeding 3 or falling below 0.5, along with a total count of sIg kappa or lambda light chain single-positive events surpassing 20, or when sIg light chain double-negative events exceeded 70%, with a total count surpassing 20. A total of 11 cases underwent this analysis. Scoring categorized seven cases as typical CLL and four cases as atypical CLL. The atypical CLL cases indicated a trend toward the increased expressions of CD20 and CD22 (Fig. S2A and B), although this was not statistically significant possibly due to the small number of cases included in the cohort.

**Table 2:** Antibodies used in the prospective analysis.

Antibody	Fluorescence	Clone	Vendor	Amount used (μL)
sIg kappa-type light chain	FITC	Rabbit Polyclonal F(ab')2/	DAKO	7.5
sIg lambda-type light chain	PE			
CD22	PerCP-Cy5.5	HIB22	BD	3.75
CD23	PECy7	M-L233	BD	3.75
ROR1	APC	2A2	Biolegend	3.75
CD79b	APC-R700	SN8	BD	3.75
CD81	APCH7	JS-81	BD	3.75
CD200	BV421	MRC OX-104	BD	3.75
CD43	BV510	1G10	BD	3.75
CD5	BV605	UCHT2	BD	3.75
CD20	BV711	2H7	BD	3.75
CD19	BV786	J25C1	BD	3.75

sIg: surface immunoglobulin.

**Figure 2:**
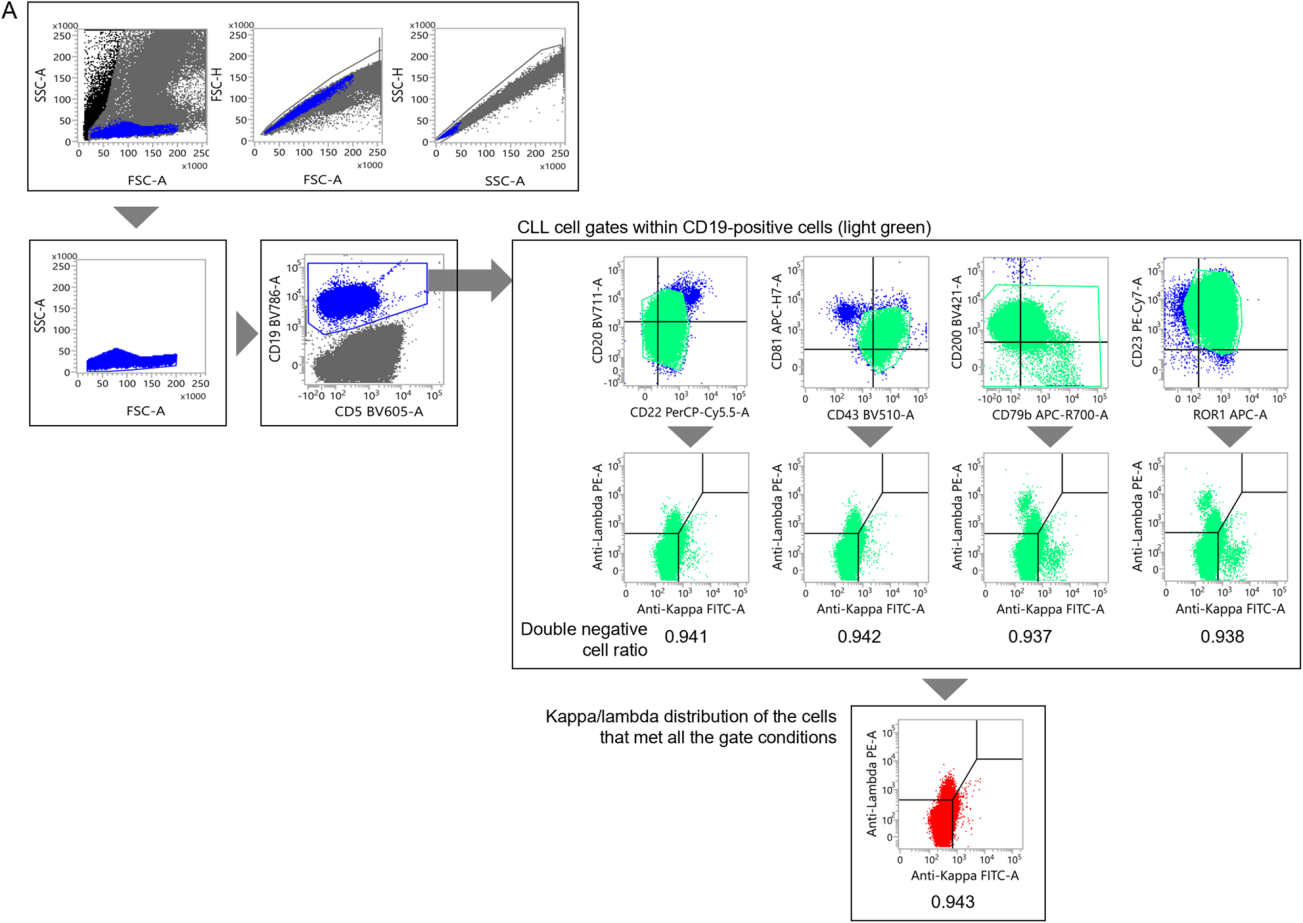

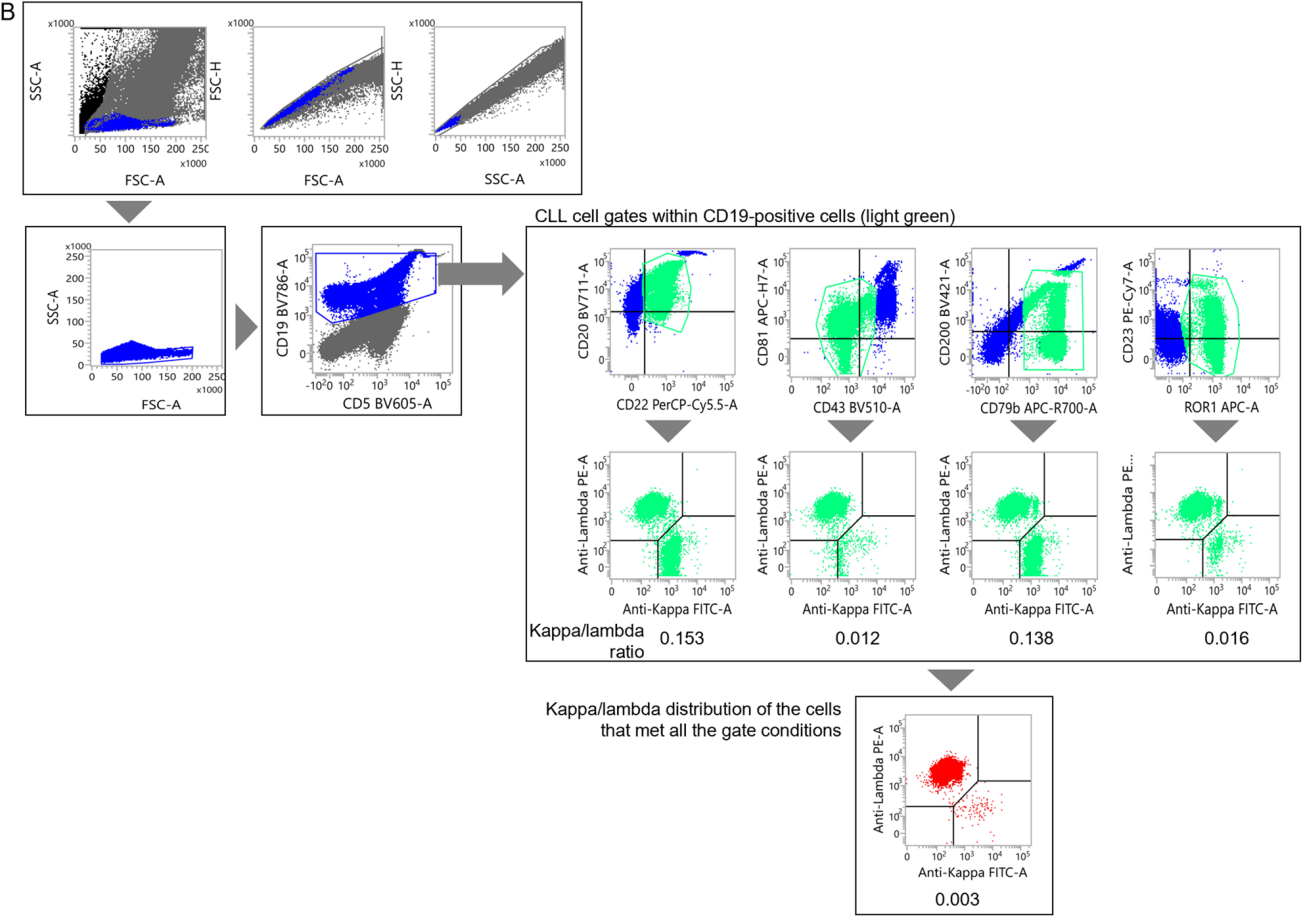

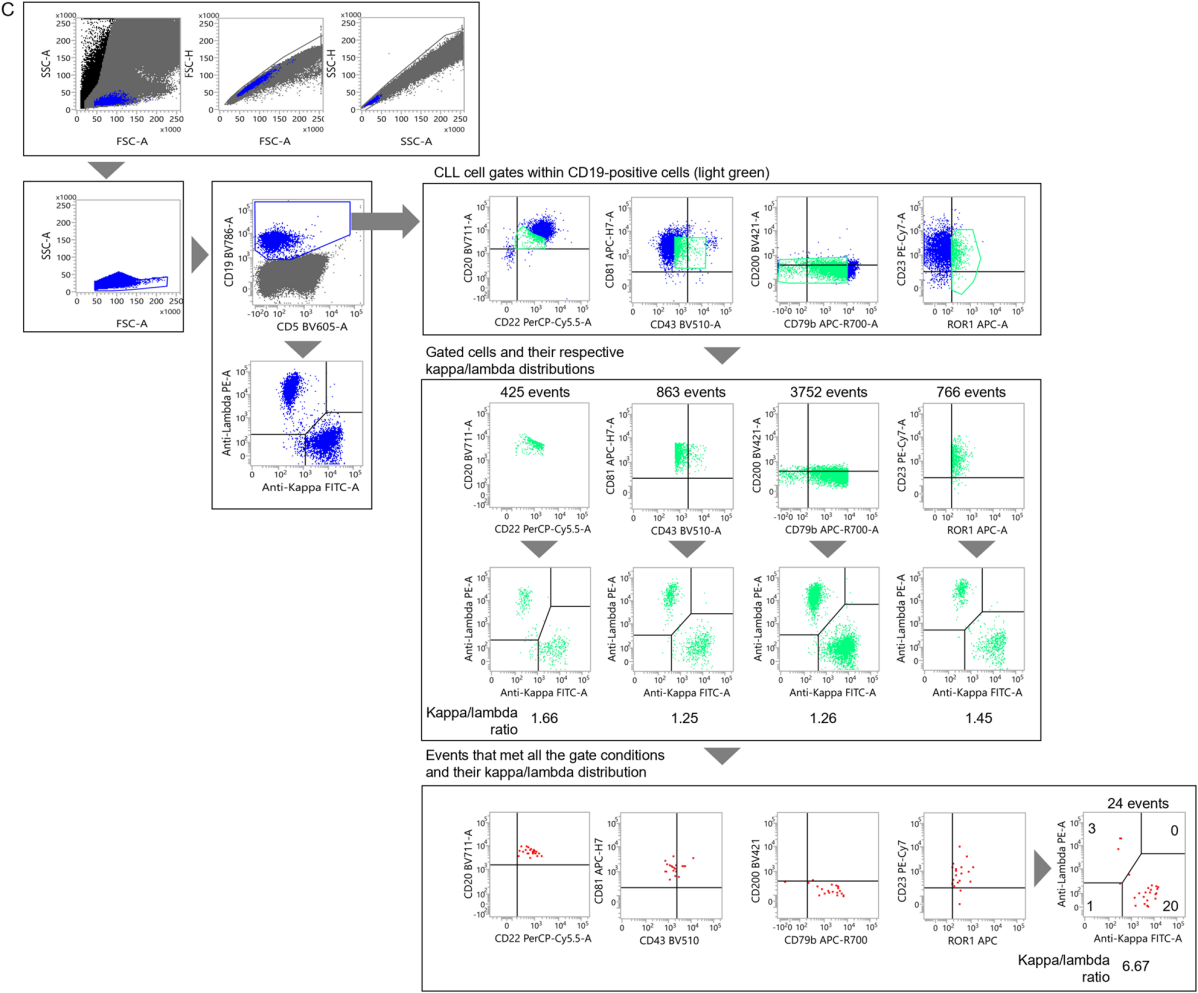
Examples of the gating process for typical and atypical CLL. (**A**, **B**) Examples of the gating process for typical CLL (**A**) and atypical CLL (**B**) that contain abundant tumor cells. (**C**) Atypical CLL with trace amount of tumor cells. The tumor cell fraction was gated by observing the sIg kappa/lambda distribution in each cell surface marker plot and the combination that met all gate conditions.

Figure 2A shows a typical CLL case with a sufficient number of tumor cells in the specimen (i.e., a case in which no gate narrowing was required). In this case, the CD22^dim^/CD20^dim^, CD43^dim^/CD81^dim^, CD79b^dim^/CD200^dim^, and ROR1^+^/CD23^+^ CLL fractions were clearly observed. Additionally, the sIg kappa/lambda light chains were attenuated in the respective gates (indicated in light green), which became slightly clearer when the kappa/lambda distribution was evaluated in the CLL fraction that met all the gate conditions (indicated in red).

Figure 2B presents a case of atypical CLL with a sufficient number of tumor cells in the specimen. In this case, the CD22^dim^/CD20^+^, CD43^−^/CD81^dim^, CD79b^+^/CD200^dim^, and ROR1^+^/CD23^dim^ CLL populations were observed, with CD23, CD81, and CD200 being widely distributed. When gating on the predicted tumor cell regions in each plot, a bias of sIg light chains toward the lambda type was evident in each development plot (kappa/lambda ratio: 0.012 to 0.153; indicated in light green). Furthermore, by applying the combination of gates displaying only those cells that met all the surface marker gate conditions, more than 99.5% of the gated cells expressed sIg light chain of the lambda type (kappa/lambda ratio: 0.003; indicated in red).

Figure 2C is an illustrative example of atypical CLL with a limited number of tumor cells in the specimen. This example has significant relevance in the context of the MRD measurements conducted in this study. In this case, no inherent bias existed in the sIg light chains across the B cell region; each development plot for the gate possibly containing CLL cells did not show a bias in the sIg light chains (kappa/lambda ratio: 1.25 to 1.66, plotted in light green). However, when the gates were set to be small enough and empirically adjusted, cells that met all the gated regions showed a bias toward kappa-type in sIg light chain (kappa/lambda ratio: 6.67, plotted in red). The number of events that satisfied all of these gated regions was as low as 20 out of a total of 500,000 events, estimating the tumor cell abundance ratio to be around 0.004%. When a similar approach was applied to investigate the bias in sIg kappa/lambda light chains in another atypical CLL case, the ratio remained within the reference range even after various gate adjustments (data not shown). This particular case was considered to be associated with the number of tumor cells falling below the lower detection limit.

### Sensitivity of MRD detection using the distribution changes of sIg light chains as an indicator

**Figure 3:**
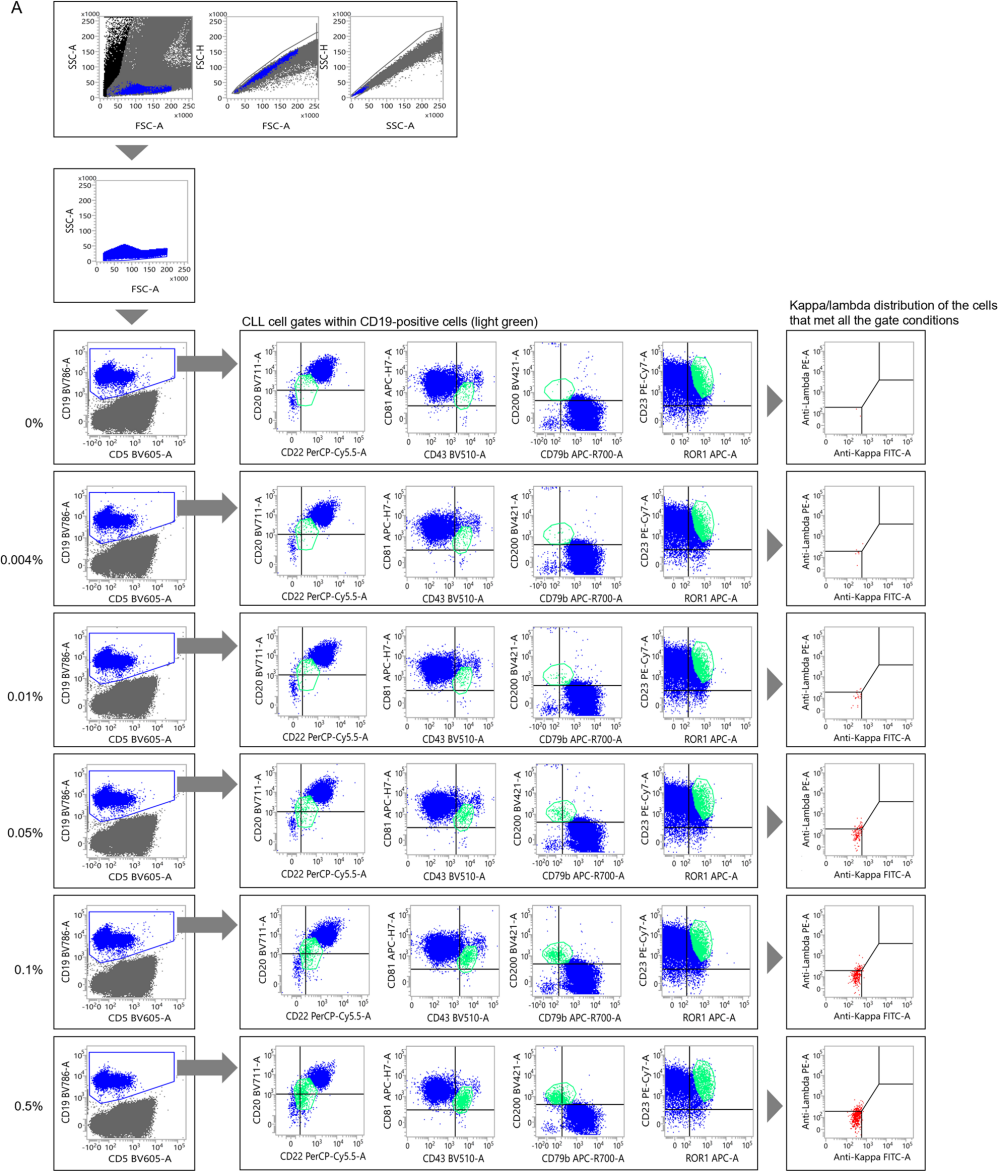

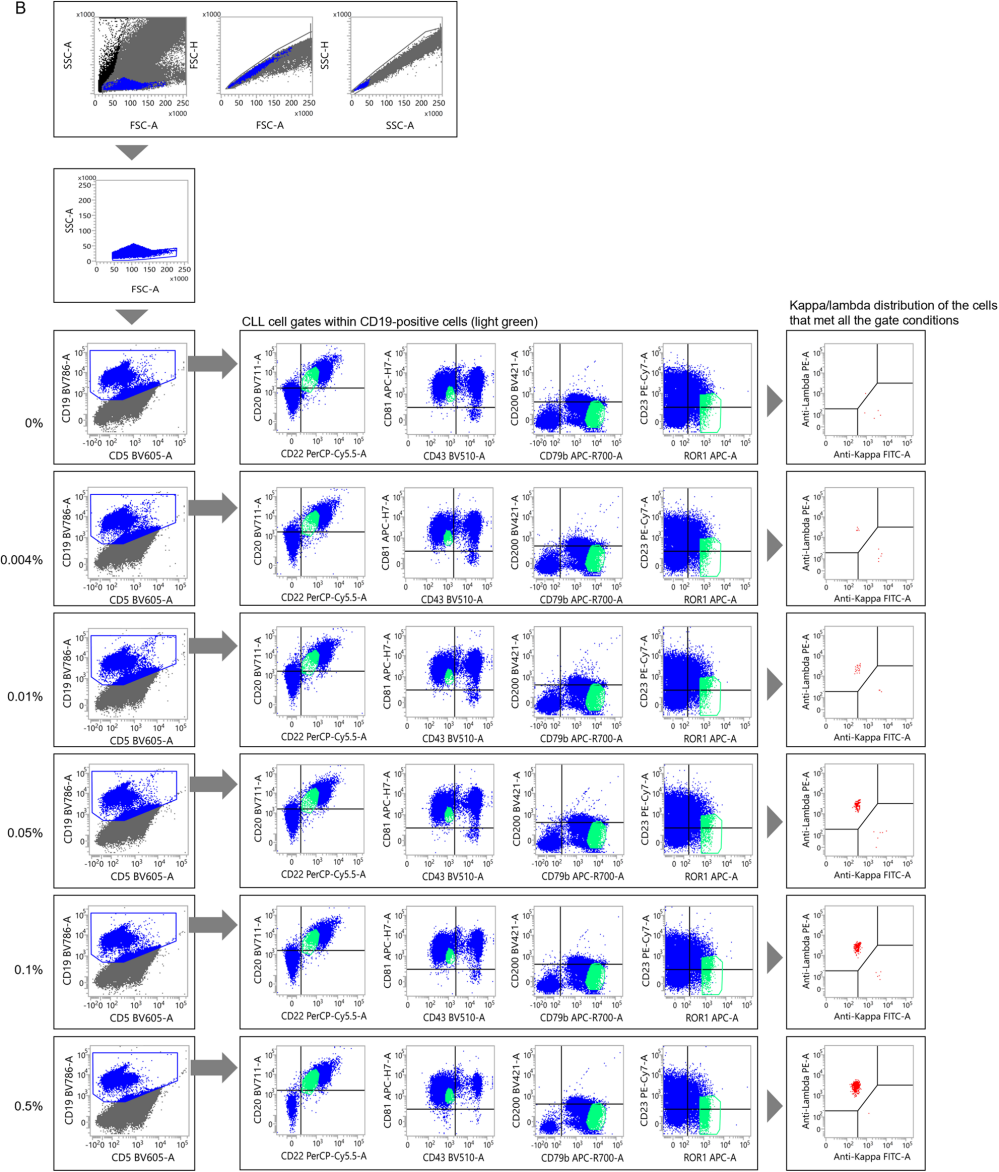
Assessment of the limit of detection for CLL cells. (**A**, **B**) Changes in the sIg light chain distribution of non-CLL specimens containing small numbers of typical CLL cells (**A**) and atypical CLL cells (**B**). The gate was narrowed down while observing the expression patterns of sIg kappa/lambda light chains. See Fig. S2A and B, in which changes in the sIg light chain were assessed without adjusting the gate but with the gate set in the pre-treatment specimen, for comparison.

A small number of CLL cells were then added to non-CLL specimens to assess the sensitivity of MRD detection using the distribution changes of sIg light chains. The results for typical CLL are presented in Figs. 3A and S3A. Initially, an undiluted specimen from a patient was gated on to select CLL cells (Fig. 2A). In this case, approximately 94% of the sIg light chains were plotted in the region where both sIg kappa/lambda light chains were attenuated. The gating setup used in this process was then applied to a non-CLL peripheral blood sample to which the CLL cells were added (Fig. S3A). In this experiment, changes in the distribution of sIg light chains, specifically a significantly increased number of cells with attenuated expression, were observed in specimens with a CLL cell rate of 0.01% or higher when compared to specimens without added tumor cells (Table S1). Furthermore, when the gating region was narrowed down while inspecting the distribution of sIg light chains to search for regions where sIg light chains were not expressed (Fig. 3A), an increase in such cells became clearer as the percentage of tumor cells increased (Table 3). However, statistical analysis was not performed, because the merged gate used for the specimen without CLL cells did not contain a sufficient number of cells.

**Table 3:** Events distributed to each sIg light chain fraction for typical CLL (narrowed gate).

CLL cell proportion in diluted specimen (%)	Lambda	Kappa	Double negative	Double positive	Total
0	0	0	2	0	2
0.004	2	1	5	0	8
0.01	3	0	20	0	23
0.05	27	5	101	0	133
0.1	44	10	228	0	282
0.5	76	13	457	0	546

sIg: surface immunoglobulin.

A similar study was conducted using non-CLL peripheral blood mixed with atypical CLL cells. The tumor cells from an atypical CLL patient used in this study were gated on to specifically select CLL cells (Fig. 2B). The sIg light chains were predominantly composed of lambda chains in approximately 100% of the gated cells. When the gating setup for the undiluted specimen from the CLL patient was applied to a normal peripheral blood specimen to which diluted CLL cells were added, the kappa/lambda ratio of sIg light chains in the specimen with 0.1% or more CLL cells was significantly lower than that without CLL cells, but the ratio did not surpass our threshold of more than 3 or less than 0.5 (Fig. S3B and Table S2). Further adjustments were made to the gating region (Fig. 3B). By fine-tuning the gates by determining regions where sIg light chains were biased toward kappa or lambda, the kappa/lambda ratio was found to be less than 0.5 in specimens with more than 0.01% tumor cells present (Table 4). The Chi-square test confirmed that the changes in the ratio were significant in these specimens when compared to the specimen without tumor cells added. Collectively, we concluded that MRD at the 10^–4^ level can be detected, even in atypical CLL patients, by strictly defining the region containing tumor cells and confirming the bias of the sIg light chains.

**Table 4:** Events distributed to each sIg light chain fraction for atypical CLL (narrowed gate).

CLL cell proportion in diluted specimen (%)	Lambda	Kappa	Double negative	Double positive	Total	p-value*
0	1	4	0	0	5	
0.004	6	5	0	0	11	0.197
0.01	23	3	0	0	26	0.001
0.05	129	6	0	0	135	1.18E−10
0.1	241	5	0	0	246	1.65E−20
0.5	631	1	0	0	632	2.63E−90

sIg: surface immunoglobulin.

*p-values calculated by Chi-square test in comparison to samples with 0% CLL cells.

## Discussion

With significant advancements have been made in CLL treatment, more CLL patients have achieved remission, leading to the nearly complete disappearance of tumor cells from the peripheral blood^31,32^. Consequently, the measurement of MRD can be crucial for accurately predicting prognosis and determining the need for re-treatment^15^. Thanks to the concerted efforts of research groups like ERIC, MRD measurement in CLL has been thoroughly validated, standardized, and harmonized^27^. However, for atypical CLL cases, MRD validation still necessitates pre-treatment information on cell surface markers, and the identification of the optimal MRD measurement method remains a priority for such instances. Surface markers such as CD200 and ROR1 have demonstrated utility in diagnosing atypical CLL^23,33^, yet even with these markers, distinguishing CLL cells from normal B cells can pose challenges, complicating MRD measurement. In this study, we showed that MRD could be detected with high sensitivity, even in atypical CLL, by utilizing a method of precise gating using the kappa/lambda ratio of sIg light chains as a reference. In our experience, atypical CLL tends to express sIg light chains more prominently than typical CLL, whereas typical CLL exhibits different surface markers from normal B lymphocytes, such as weakly positive CD5 and loss of CD79b, allowing for the detection of tumor cells based on these surface marker differences. Given these considerations, it is practical to utilize MRD measurement parameters tailored to each specimen in clinical settings, accounting for the characteristics of typical and atypical CLL. In our approach, we set the gate size as small as possible to detect changes in the kappa/lambda ratio of sIg light chains with high sensitivity. This may result in some tumor cells falling outside the designated gate, representing a limitation of MRD quantification. However, our method excels in detecting the presence of MRD through flow cytometry with the highest sensitivity. Although it is desirable to have information on CLL cell surface markers measured before treatment, the expression pattern of these markers occasionally changes upon recurrence after treatment. Atypical CLL, in particular, tends to strongly express sIg light chains^34^, making it easier to detect changes in the kappa/lambda ratio. Taking these situations into account, this approach can be applied even in the absence of pre-treatment data.

Although our approach is admittedly more complex, as it requires advanced gating skills, we propose that it is a valuable MRD detection method that can be utilized in clinical laboratories without the need for specialized reagents or devices. It is also worth noting that recent research has identified LEF-1, CD160, and CD180 as surface markers specific to, or at least, applicable to, atypical CLL^30,35,36^. It is anticipated that in the near future, a flow cytometry method applicable to a broader spectrum of CLL cases will be established.

## Materials and methods

### Samples

This study conducted an exploratory analysis using residual specimens from clinically performed flow cytometry for the detection of tumor cells in patients with CLL. The data includes samples measured in the past (retrospective cohort) and those after the start of this study (prospective cohort). Residual specimens of peripheral blood or bone marrow from CLL patients collected between May 2017 and September 2023 and provided for clinical purposes were used for this study. The diagnosis of CLL was made according to the 2018 International Workshop on CLL diagnostic criteria^7^. Because the study was designed to measure MRD, retrospective and prospective analyses included samples taken at the time of diagnosis as well as during or after therapeutic intervention, without considering the specific type of therapeutic agent used. B-cell diseases such as mantle cell lymphoma, lymphoplasmacytic leukemia, and monoclonal B-cell lymphomatosis were excluded pathologically in conjunction with immunohistochemistry, cytogenetic testing, and molecular analysis. Apart from excluding cases without CLL, no additional inclusion or exclusion criteria were applied. A total of 39 and 11 CLL cases were included in the retrospective and prospective analyses, respectively. Control (non-CLL) samples were obtained from 18 patients aged 18 years or older with non-hematopoietic diseases, no significant changes in any of the three blood count data, and no history of receiving radiation or chemotherapy that potentially affects hematopoiesis. Since this research specifically investigates the potential utility of the kappa/lambda ratio of sIg light chains detected through flow cytometry in identifying MRD in CLL under the exploratory nature, we did not estimate the sample size required for statistical verification. Specimens were collected from patients who provided comprehensive written informed consent for the use of their specimens in clinical studies directed at the National Cancer Center Hospital in Japan, and the study was conducted under the approval of the National Cancer Center Institutional Review Board (approval number: 2019–163), which oversees the ethical aspects of this research protocol. The study was also conducted in accordance with the Ethical Guidelines for Medical and Biological Research Involving Human Subjects in Japan and the Declaration of Helsinki.

### Cell preparation, staining, and flow cytometry analysis

Freshly prepared specimens were used for the data acquisition. Density gradient centrifugation was not applied for cell separation; instead, whole blood cells were used for data acquisition. After calculating the leukocyte count using an automated hematology analyzer, peripheral blood and bone marrow specimens were divided into aliquots to obtain a leukocyte count of 5 million per tube and washed three times with phosphate-buffered saline (PBS). During the washing process throughout the experiments, 2 mL of PBS was added to the samples, followed by vortexing and centrifugation at 500 × G for 5 min, and then the supernatant was aspirated. After washing, cells were resuspended in 1 mL of PBS, adjusted so the sample had a leukocyte count of 5000/µL, and dispensed at 100 µL (for retrospective analysis) or 150 µL (for prospective analysis) per tube. Tables 1 and 2 indicate the combinations and amounts of antibodies used for the retrospective and prospective analyses, respectively. After 15 min of incubation in the dark at room temperature, hemolysis and washing were performed using a BD FACS™ Lyse Wash Assistant (BD, NJ, USA) following the instrument's operating instructions. BD FACSCanto™ II (BD) and BD FACSLyric™ (BD) were used for data acquisition for retrospective and prospective analyses, respectively.

In the retrospective analysis, the positivity of surface markers was determined for the CD19-positive B cell region within CD45-positive cells. Surface markers of 50,000 cells per specimen was obtained in this analysis. For the prospective analysis, the determination was made for CD19-positive B cells within the area identified as lymphocytes by FSC-A/SSC-A, with surface markers of 500,000 cells per specimen being obtained. An isotype control cutoff was set at the position where the positivity rate of each fluorophore in the gated region was less than 5%. The percentage of CD19-positive cells with signal intensity above the cutoff value was defined as the positivity rate^16^. Three levels of criteria were applied, with > 80% defined as positive, > 20–80% as weakly positive (diminished, or dim), and ≤ 20% as negative^30^. In this study, the following criteria were used to distinguish between typical and atypical CLL: a total score was first determined by allocating one point to each of the following characteristics: CD5 positivity, CD23 positivity, CD79b weak expression or negativity, and smIg weak expression or negativity, each of which is commonly associated with typical CLL^34,37^. A total score of 3 or 4 points was designated as indicative of typical CLL, while a score of two points indicated atypical CLL.

### Estimation of the assay sensitivity of CLL cell detection in peripheral blood specimens

The detection sensitivity was assessed by spiking-in a small number of CLL cells in serial dilutions to non-CLL samples^38^. Samples from typical and atypical CLL cases with lymphocyte percentages of 61% and 28%, containing approximately 51% and 6% CLL cells as observed by microscopy, respectively, were used for the validation. To achieve CLL cell ratios of 0%, 0.004%, 0.01%, 0.05%, 0.1%, and 0.5%, peripheral blood from CLL cases was mixed with non-CLL peripheral blood and divided into tubes to achieve a leukocyte count of 1.5 million. The procedure involved staining with the 12 antibodies listed in Table 2, except that three times the amount of antibody was added. Measurements were conducted using BD FACSLyric™.

### Statistical analysis

For the distribution bias of the sIg light chain kappa/lambda ratio detected by flow cytometry, the total number of cells required to demonstrate that the kappa/lambda ratio in tumor cells exceeds 3 or is less than 0.5, assuming a tumor cell ratio of 1 in 10,000 (10^−4^) in the measurement sample, was calculated using a Poisson approximation with a significance level of 5% and a power of 80%. Consequently, we determined that approximately 62,000 events would be needed, indicating that our measurements are sufficient. In the experiments comparing the positivity and MFI of cell surface markers between the two groups, the F-test was initially utilized to assess whether the variances were equal. If the F-test indicated unequal variances (p < 0.05), Welch's *t*-test was applied. Conversely, when the F-test showed equal variances (p ≥ 0.05), the Student's *t*-test was used. To compare the number of cells assigned to each gate or fraction in the flow cytometry plots across different samples, the Chi-square test was used. Statistical significance was determined when p < 0.05.

### Supplementary Information

Supplementary Information.

## Data Availability

All data generated or analyzed during this study are included in this article and its supplementary information files.
