# Supplementary material for: The kappa/lambda ratio of surface immunoglobulin light chain as a valuable parameter for MRD assessment in CLL with atypical immunophenotype
Source: Sci Rep. 2024 Jun 11;14:13452. doi: 10.1038/s41598-024-64398-6 (PMC11166639; doi:10.1038/s41598-024-64398-6)
Supplement: Supplementary file 1 — Supplementary Information. [file 41598_2024_64398_MOESM1_ESM.pdf]

Figure S1

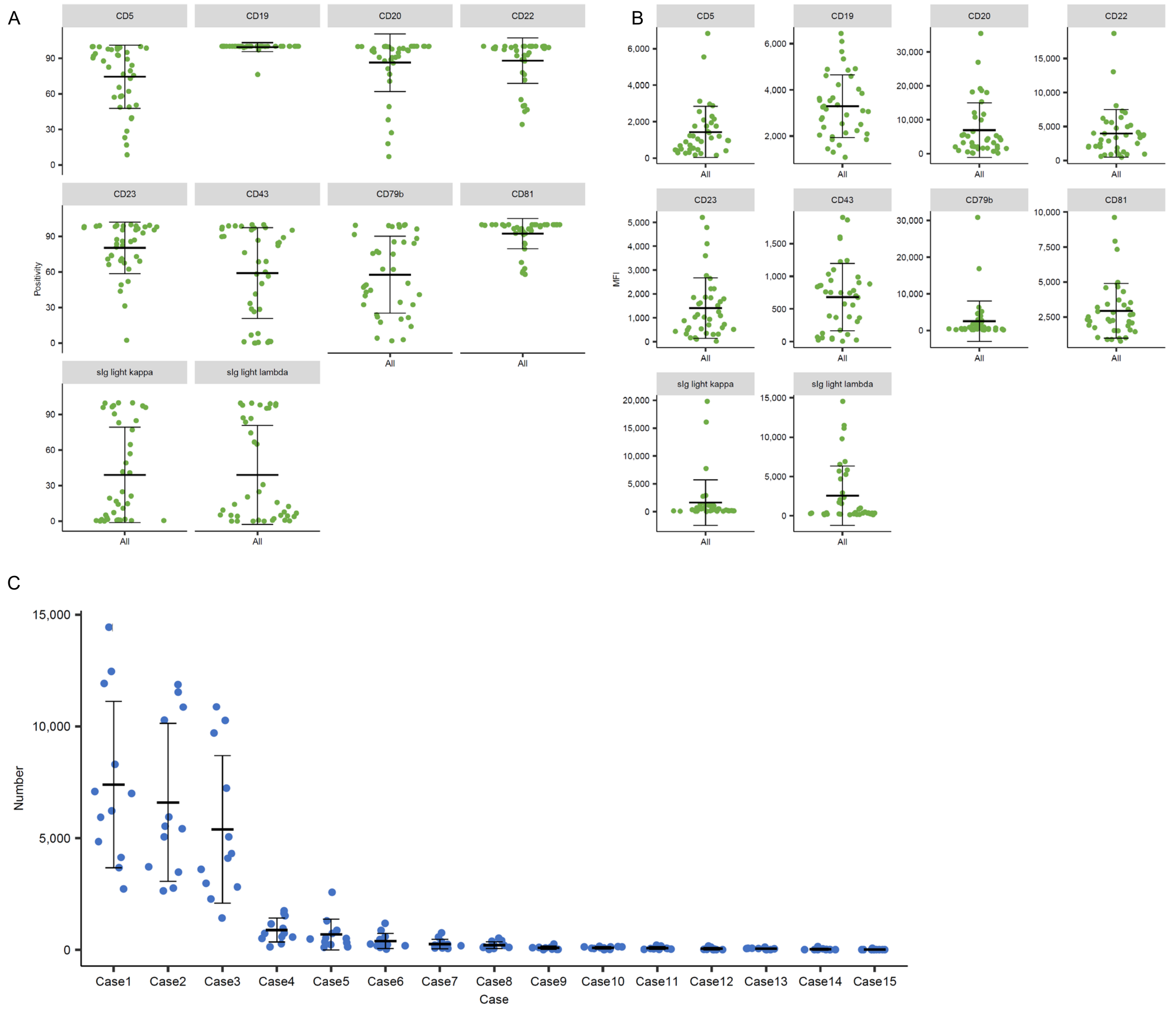

Figure S1. Variation in expression of cell surface markers between cases (retrospective study cohort).
(A and B) Positivity (A) and MFI (B) of cell surface markers measured in 39 clinically diagnosed cases of CLL. (C) Gates were placed in regions corresponding to tumor cells for each of the 15 cases of atypical CLL. Subsequently, using normal peripheral blood from 12 non-CLL donors, the number of cells included in the gate set for CLL cells was quantified. The plot shows the number of normal cells counted within the gate set for CLL cells.

Figure S2

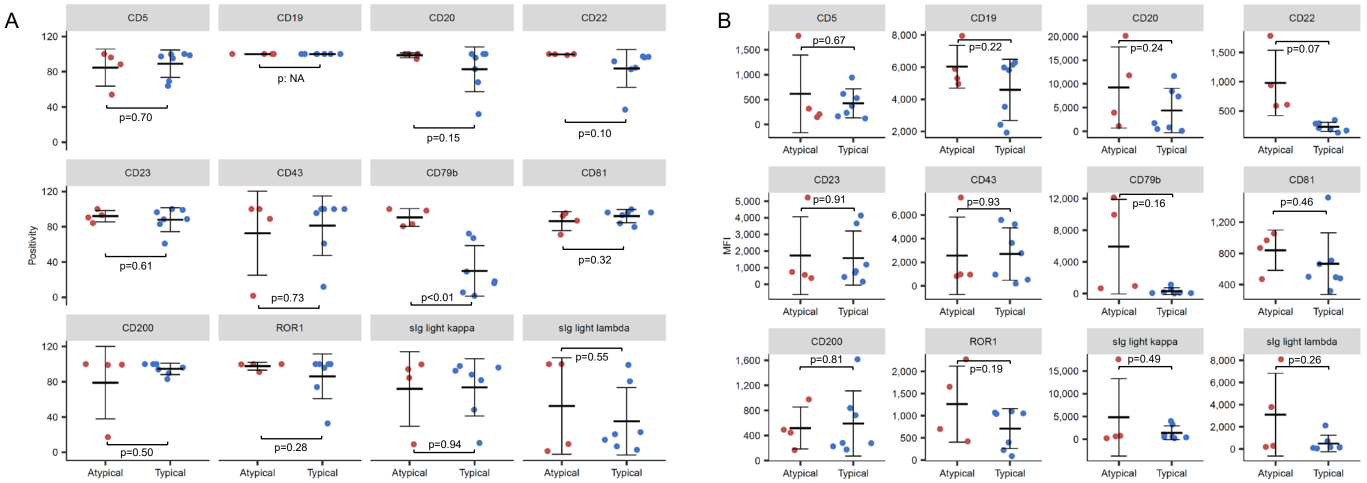

Figure S2. Variation in expression of cell surface markers between cases (prospective study cohort)
(A, B) Positivity (A) and MFI (B) of cell surface markers measured in 11 cases of CLL. P-values were calculated between the groups using Welch's *t*-test or Student's *t*-test, depending on whether the values exhibited unequal or equal variances, respectively. A p-value less than 0.05 was considered statistically significant.

Figure S3

A

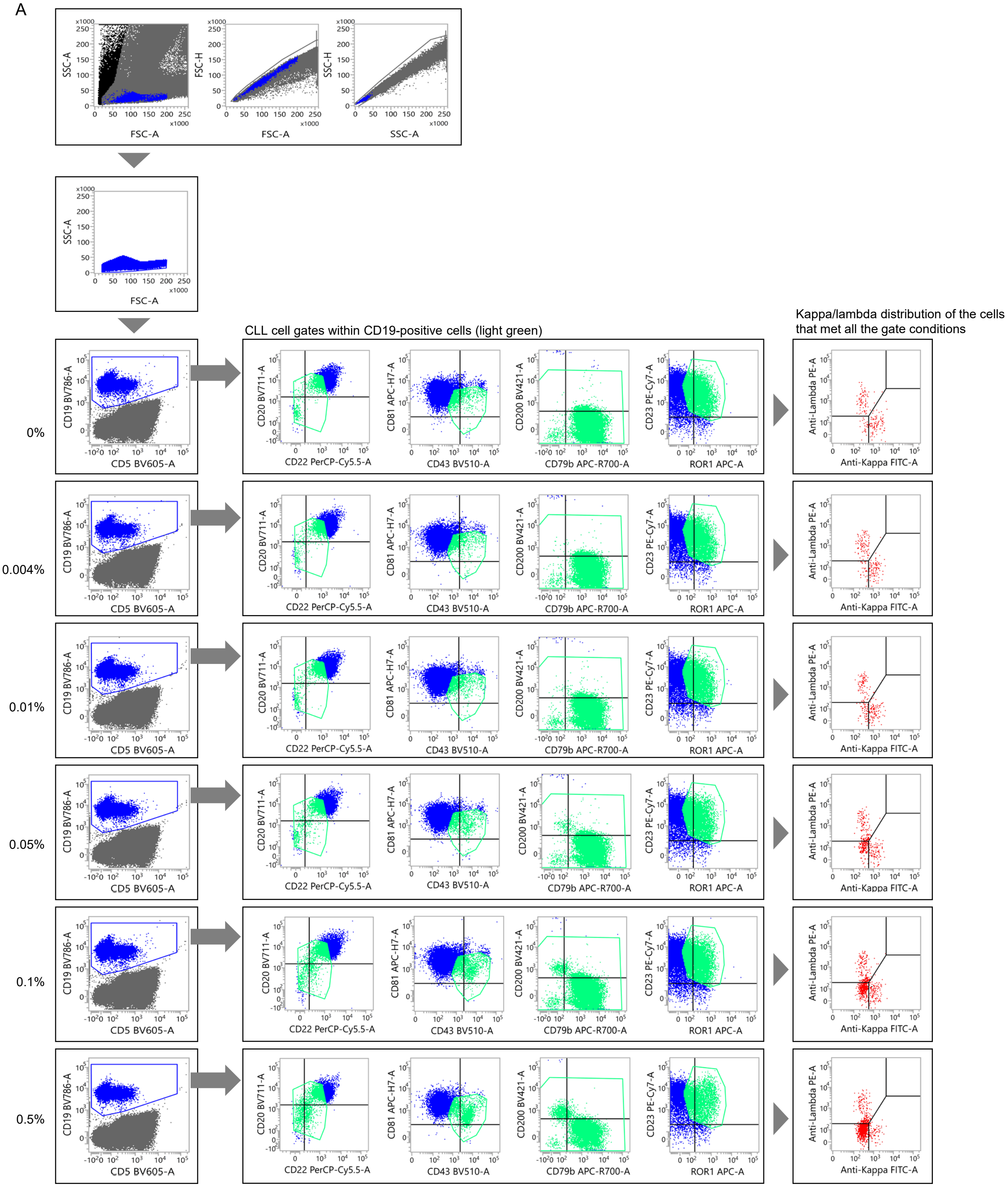

SSC-A
FSC-A
FSC-H
SSC-H
CLL cell gates within CD19-positive cells (light green)
Kappa/lambda distribution of the cells
that met all the gate conditions
0%
0.004%
0.01%
0.05%
0.1%
0.5%
CD19 BV786-A
CD5 BV605-A
CD20 BV711-A
CD22 PerCP-Cy5.5-A
CD81 APC-H7-A
CD43 BV510-A
CD200 BV421-A
CD79b APC-R700-A
CD23 PE-Cy7-A
ROR1 APC-A
Anti-Lambda PE-A
Anti-Kappa FITC-A

Figure S3

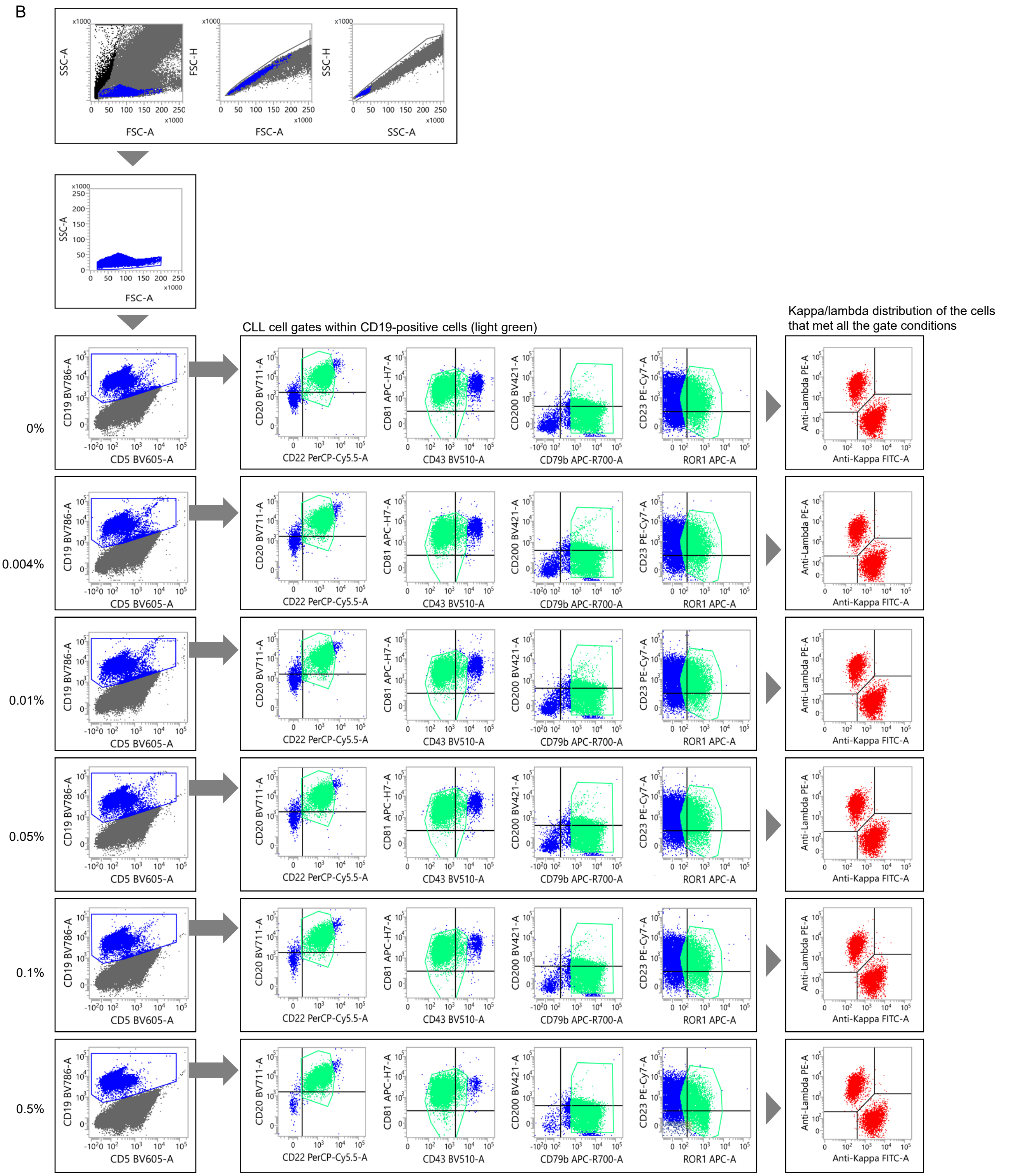

Figure S3. Assessment of the limit of detection for CLL cells.
(A, B) Changes in sIg light chain distribution of non-CLL specimens containing small numbers of typical CLL cells (A) and atypical CLL cells (B). sIg light chain distributions were evaluated without adjusting the gate but with the gate set in the pre-treatment specimen. See Fig. 3 for comparison. sIg: surface immunoglobulin.

Table S1. Events distributed to each sIg light chain fraction for typical CLL (original gate)

CLL cell proportion in diluted specimen	Lambda	Kappa	Double negative	Double positive	Total	p-value*
0%	82	97	26	0	205	
0.004%	95	101	30	0	226	0.861
0.01%	82	107	58	0	247	0.012
0.05%	129	114	154	0	397	8.66E-11
0.1%	171	130	348	0	649	2.82E-25
0.5%	221	92	812	0	1125	2.56E-69

*p-values calculated by chi-square test in comparison to samples with 0% CLL cells

sIg: surface immunoglobulin

Table S2. Events distributed to each sIg light chain fraction for aypical CLL (original gate)

CLL cell proportion in diluted specimen	Lambda	Kappa	Double negative	Double positive	Total	p-value*
0%	2890	3954	7	2	6853	
0.004%	2946	3817	3	5	6771	0.148
0.01%	2683	3691	4	3	6381	0.820
0.05%	2938	3729	5	1	6673	0.154
0.1%	3014	3700	4	10	6728	1.00E-03
0.5%	4503	3205	6	8	7722	8.24E-83

*p-values calculated by chi-square test in comparison to samples with 0% CLL cells

sIg: surface immunoglobulin
